# Absence of Nucleotide-Oligomerization-Domain-2 Is Associated with Less Distinct Disease in *Campylobacter jejuni* Infected Secondary Abiotic IL-10 Deficient Mice

**DOI:** 10.3389/fcimb.2017.00322

**Published:** 2017-07-13

**Authors:** Markus M. Heimesaat, Ursula Grundmann, Marie E. Alutis, André Fischer, Stefan Bereswill

**Affiliations:** Department of Microbiology and Hygiene, Charité—University Medicine Berlin Berlin, Germany

**Keywords:** *Campylobacter jejuni*, nucleotide-oligomerization-domain-2 (Nod2), IL-10^−/−^ mice, secondary abiotic (gnotobiotic) mice, IL-23/IL-22/IL-18 axis, pro-inflammatory immune responses, bacterial translocation, colonization resistance

## Abstract

Human *Campylobacter jejuni*-infections are progressively increasing worldwide. Despite their high prevalence and socioeconomic impact the underlying mechanisms of pathogen-host-interactions are only incompletely understood. Given that the innate immune receptor nucleotide-oligomerization-domain-2 (Nod2) is involved in clearance of enteropathogens, we here evaluated its role in murine campylobacteriosis. To address this, we applied Nod2-deficient IL-10^−/−^ (Nod2^−/−^ IL-10^−/−^) mice and IL-10^−/−^ counterparts both with a depleted intestinal microbiota to warrant pathogen-induced enterocolitis. At day 7 following peroral *C. jejuni* strain 81–176 infection, Nod2 mRNA was down-regulated in the colon of secondary abiotic IL-10^−/−^ and wildtype mice. Nod2-deficiency did neither affect gastrointestinal colonization nor extra-intestinal and systemic translocation properties of *C. jejuni*. Colonic mucin-2 mRNA was, however, down-regulated upon *C. jejuni*-infection of both Nod2^−/−^ IL-10^−/−^ and IL-10^−/−^ mice, whereas expression levels were lower in infected, but also naive Nod2^−/−^ IL-10^−/−^ mice as compared to respective IL-10^−/−^ controls. Remarkably, *C. jejuni*-infected Nod2^−/−^ IL-10^−/−^ mice were less compromised than IL-10^−/−^ counterparts and displayed less distinct apoptotic, but higher regenerative cell responses in colonic epithelia. Conversely, innate as well as adaptive immune cells such as macrophages and monocytes as well as T lymphocytes and regulatory T-cells, respectively, were even more abundant in large intestines of Nod2^−/−^ IL-10^−/−^ as compared to IL-10^−/−^ mice at day 7 post-infection. Furthermore, IFN-γ concentrations were higher in *ex vivo* biopsies derived from intestinal compartments including colon and mesenteric lymph nodes as well as in systemic tissue sites such as the spleen of *C. jejuni* infected Nod2^−/−^ IL-10^−/−^ as compared to IL10^−/−^ counterparts. Whereas, at day 7 postinfection anti-inflammatory IL-22 mRNA levels were up-regulated, IL-18 mRNA was down-regulated in large intestines of Nod2^−/−^ IL-10^−/−^ vs. IL-10^−/−^ mice. In summary, *C. jejuni*-infection induced less clinical signs and apoptosis, but more distinct colonic pro- and (of note) anti-inflammatory immune as well as regenerative cell responses in Nod2 deficient IL-10^−/−^ as compared to IL-10^−/−^ control mice. We conclude that, even though colonic Nod2 mRNA was down-regulated upon pathogenic challenge, Nod2-signaling is essentially involved in the well-balanced innate and adaptive immune responses upon *C. jejuni*-infection of secondary abiotic IL-10^−/−^ mice, but does neither impact pathogenic colonization nor translocation.

## Introduction

Host immune responses are essential for controlling and combating enteropathogenic infections. The nucleotide-oligomerization-domain (Nod)-like receptors belong to a family of intracellular pattern recognition receptors that sense microbial products and damage-associated factors thereby regulating host innate immune responses (Shaw et al., 2008). The Nod2 receptor is expressed by innate (including dendritic cells, macrophages, and monocytes) and adaptive (such as T lymphocytes) immune cell populations as well as by Paneth cells (Ogura et al., 2001, 2003; Hisamatsu et al., 2003; Tada et al., 2005). Upon activation by muramyl dipeptide (MDP) comprizing a major constituent of bacterial peptidoglycans and known for its immunomodulatory properties (Inohara and Nunez, 2003), Nod2 signaling confers resistance against a multitude of bacterial species including enteropathogens (Girardin et al., 2003a,b; Shaw et al., 2008; Grimes et al., 2012). Among these, *Campylobacter jejuni* can be found as commensal bacteria colonizing the gastrointestinal tract of wild and domestic animals. Humans become mainly infected by ingestion of *C. jejuni* via contaminated products derived from livestock animals or by pathogen-containing surface water (Guerry and Szymanski, 2008; Lane and Martin, 2012). Infected individuals may be asymptomatic or display symptoms of varying degree depending on their immune status and on the virulence of the respective bacterial strain. Whereas, some patients might complain about mild malaise with watery diarrhea, others present with severe symptoms including fever, abdominal cramps, and ulcerative enterocolitis with inflammatory bloody diarrhea requiring antibiotic treatment and hospitalization in immunocompromised patients (Kist and Bereswill, 2001; Backert et al., 2017). Histopathological changes of affected intestinal tissues are characterized by ulcerations, crypt abscesses, and elevated immune cell numbers in the colon (van Spreeuwel et al., 1985; Walker et al., 1986; Kist and Bereswill, 2001). In the vast majority of cases disease is self-limiting, but post-infectious sequelae affecting the nervous system (i.e., Guillain-Barré syndrome, Miller Fisher syndrome, and Bickerstaff encephalitis), the joints (i.e., reactive arthritis), and the gastrointestinal tract (i.e., irritable bowel syndrome) might develop with a latency of weeks to months postinfection (p.i.) as reviewed by Backert et al. (2017). Whereas, human *C. jejuni* infections are progressively increasing worldwide in both developed and developing countries (Backert et al., 2017), the molecular mechanisms underlying host-pathogen interactions are only incompletely understood. One of the reasons is that for a long time appropriate *in vivo* models were simply not available (Masanta et al., 2013). Conventionally colonized mice, for instance, display a strong physiological colonization resistance preventing from stable enteropathogenic colonization (Bereswill et al., 2011; Fiebiger et al., 2016). Following depletion of the intestinal microbiota by broad-spectrum antibiotic treatment, however, secondary abiotic (i.e., gnotobiotic) IL-10^−/−^ mice can be stably infected by the pathogen with high loads and develop non-selflimiting ulcerative enterocolitis with bloody diarrhea, thus displaying key features of campylobacteriosis in immunocompromised patients (Haag et al., 2012; Heimesaat et al., 2014a,c; Fiebiger et al., 2016). In the present study we therefore applied the secondary abiotic murine IL-10^−/−^ infection model to further elucidate the impact of Nod2 in *C. jejuni*-host interactions.

## Materials and methods

### Ethics statement

All animal experiments were conducted according to the European Guidelines for animal welfare (2010/63/EU) with approval of the commission for animal experiments headed by the “Landesamt für Gesundheit und Soziales” (LaGeSo, Berlin, registration number G0135/10). Animal welfare was monitored twice daily by assessment of clinical conditions.

### Generation of secondary abiotic mice and *C. jejuni* infection

Female IL-10^−/−^ mice and IL-10^−/−^ mice lacking Nod2 (Nod2^−/−^ IL-10^−/−^) mice (all in C57BL/6j background) were bred, raised, and kept within the same specific pathogen free (SPF) unit of the Forschungseinrichtungen für Experimentelle Medizin (FEM, Charité—University Medicine Berlin). To counteract physiological colonization resistance and assure stable intestinal colonization of the pathogen (Bereswill et al., 2011), secondary abiotic mice (i.e., gnotobiotic) virtually lacking an intestinal microbiota were generated by broad-spectrum antibiotic treatment for 8 weeks as described previously (Heimesaat et al., 2006; Bereswill et al., 2011; Haag et al., 2012). In brief, immediately post weaning 3 weeks old mice were subjected to an 8 weeks course of broad-spectrum antibiotic treatment by adding ampicillin plus sulbactam (1 g/L; Ratiopharm, Germany), vancomycin (500 mg/L; Cell Pharm, Germany), ciprofloxacin (200 mg/L; Bayer Vital, Germany), imipenem (250 mg/L; MSD, Germany), and metronidazole (1 g/L; Fresenius, Germany) to the autoclaved drinking water (*ad libitum*). Three days prior infection, the antibiotic cocktail was withdrawn and replaced by autoclaved tap water. Mice (3 months of age) were then perorally infected with 10^9^ colony forming units (CFU) of viable *C. jejuni* strain 81−176 (kindly provided by Prof. Dr. Steffen Backert, University of Erlangen-Nuremberg, Germany) in a volume of 0.3 mL phosphate buffered saline (PBS; Gibco, life technologies, UK) on 2 consecutive days (days 0 and 1) by gavage as described earlier (Bereswill et al., 2011). To prevent mice from contaminations, animals were continuously maintained in a sterile environment (autoclaved food and drinking water or sterile antibiotic cocktail) and handled under strict aseptic conditions.

### Clinical conditions

To assess clinical signs of *C. jejuni* induced infection on a daily basis, a standardized cumulative clinical score (maximum 12 points), addressing the occurrence of blood in feces (0: no blood; 2: microscopic detection of blood by the Guajac method using Haemoccult, Beckman Coulter/PCD, Germany; 4: macroscopic blood visible), diarrhea (0: formed feces; 2: pasty feces; 4: liquid feces), and the clinical aspect (0: normal; 2: ruffled fur, less locomotion; 4: isolation, severely compromised locomotion, pre-final aspect) was used as described earlier (Haag et al., 2012; Alutis et al., 2015a,b).

### Sampling procedures and histopathology

Mice were sacrificed at day 7 postinfection (p.i.) by isofluran treatment (Abbott, Germany). Colonic *ex vivo* biopsies were asserved under sterile conditions and collected from each mouse in parallel for microbiological, histopathological, immunohistopathological, and immunological analyses. Histopathological changes were determined in samples derived from the colon that were immediately fixed in 5% formalin and embedded in paraffin. Sections (5 μm) were stained with hematoxylin and eosin (H&E), examined by light microscopy (magnification 100 × and 400 ×) and histopathological changes quantitatively assessed applying respective histopathological scoring systems (maximum 4 points) as described previously (Heimesaat et al., 2014a).

### Immunohistochemistry

*In situ* immunohistochemical analysis of colonic paraffin sections was performed as stated elsewhere (Heimesaat et al., 2010, 2014c; Alutis et al., 2015a,b). For each animal, the average number of positively stained cells within at least six high power fields (HPF, 0.287 mm^2^, 400 × magnification) were determined microscopically by a blinded independent investigator.

### Quantitative analysis of bacterial colonization

Viable *C. jejuni* were quantitatively assessed in feces over time p.i. and in homogenates of *ex vivo* biosies taken from mesenteric lymph nodes (MLN), spleen, liver, and kidney at time of necropsy (i.e., day 7 p.i.) by culture as described earlier (Bereswill et al., 2011; Heimesaat et al., 2013). The detection limit of viable pathogens was ≈100 CFU per g.

### Cytokine detection in supernatants of intestinal and extra-intestinal *ex vivo* biopsies

Colonic *ex vivo* biopsies were cut longitudinally and washed in PBS. MLN, spleen, or strips of ~1 cm^2^ colonic tissue were placed in 24-flat-bottom well-culture plates (Nunc, Germany) containing 500 μL serum-free RPMI 1,640 medium (Gibco, life technologies, UK) supplemented with penicillin (100 U/mL) and streptomycin (100 μg/mL; PAA Laboratories, Germany). After 18 h at 37°C, culture supernatants were tested for IFN-γ, TNF, MCP-1, and IL-6 by the Mouse Inflammation Cytometric Bead Assay (CBA; BD Biosciences, Germany) on a BD FACSCanto II flow cytometer (BD Biosciences). Nitric oxide (NO) was measured by Griess reaction as described earlier (Heimesaat et al., 2006), whereas protein concentrations were determined with the Quant-iT™ Protein Assay Kit (Thermo Fisher Scientific, Germany) according to the manufacturer's instructions.

### Real-time PCR

RNA was isolated from snap frozen colonic *ex vivo* biopsies, reverse transcribed, and analyzed as described previously (Munoz et al., 2009). Murine Nod2, mucin-2 (MUC2), IFN-γ, TNF, IL-17A, IL-1β, IL-23p19, IL-22, and IL-18 mRNA expression levels were analyzed using Light Cycler Data Analysis Software (Roche, Switzerland). The mRNA of the housekeeping gene for hypoxanthine-phosphoribosyltransferase (HPRT) was used as reference, and the mRNA expression levels of the individual genes were normalized to the lowest measured value and expressed as fold expression (Arbitrary Units).

### Statistical analysis

Medians and levels of significance were determined using Mann–Whitney test (GraphPad Prism v5, USA) as indicated. Two-sided probability (*p* ≤ 0.05 were considered significant.

## Results

### Colonic Nod2 expression in *C. jejuni* strain 81–176 infected secondary abiotic IL-10^−/−^ mice

**Figure 1:**
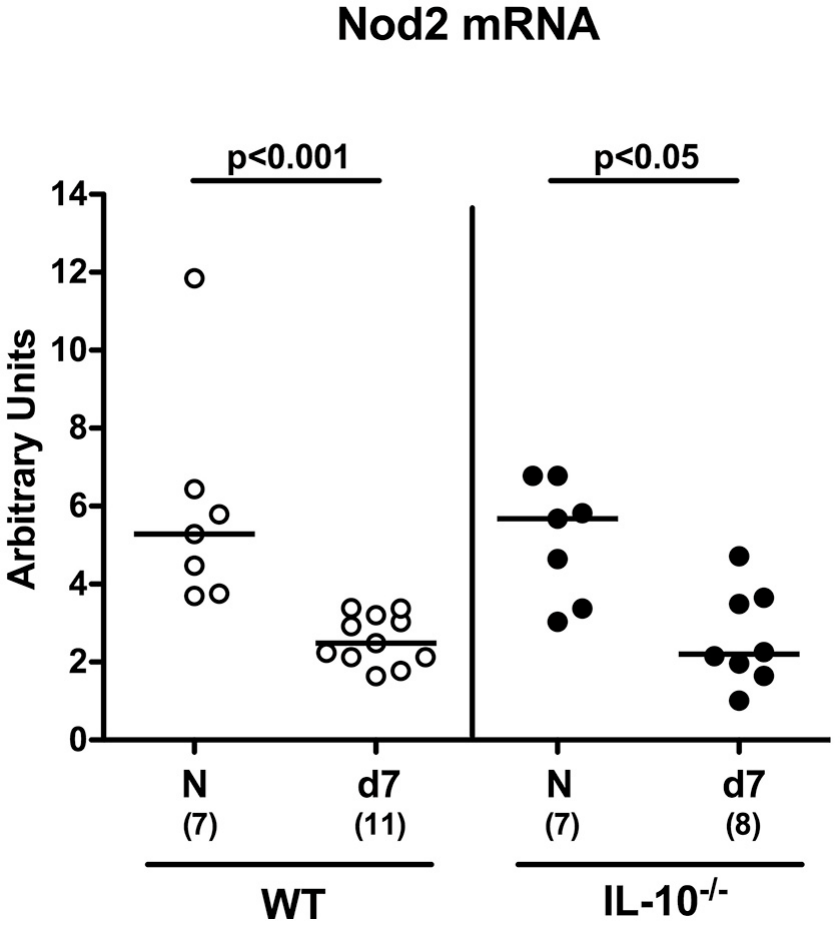
Colonic Nod2 expression in *C. jejuni* strain 81–176 infected secondary abiotic IL-10^−/−^ mice. Secondary abiotic wildtype (WT; white circles) and IL-10^−/−^ mice (black circles) were generated by broad-spectrum antibiotic treatment and perorally infected with *C. jejuni* strain 81–176 by gavage at day (d) 0 and d1. Nod2 mRNA levels were determined in colonic *ex vivo* biopsies at day 7 post-infection by Real Time PCR and expressed as Arbitrary Units (fold expression). Naive (N) mice served as uninfected controls. Medians (black bars), levels of significance (*p*-values) determined by Mann–Whitney *U*-test and numbers of analyzed animals (in parentheses) are indicated. Data were pooled from two independent experiments.

Secondary abiotic mice (namely wildtype (WT) mice, IL-10^−/−^ mice and IL-10^−/−^ mice lacking Nod2 (Nod2^−/−^ IL-10^−/−^) were generated by an 8 weeks course of broad-spectrum antibiotic treatment and colonic Nod2 expression determined following *C. jejuni* infection of WT and IL-10^−/−^ mice. In the basal state, large intestinal Nod2 mRNA levels were comparable in the large intestines of mice irrespective of their genotype (Figure 1). Seven days following peroral infection with 10^9^ CFU *C. jejuni* strain 81–176 on two consecutive days (i.e., days 0 and 1), however, Nod2 was down-regulated in the large intestines of both WT and IL-10^−/−^ mice (*p* < 0.001 and *p* < 0.05, respectively; Figure 1).

### Colonization and translocation properties of *C. jejuni* in secondary abiotic mice lacking Nod2

**Figure 2:**
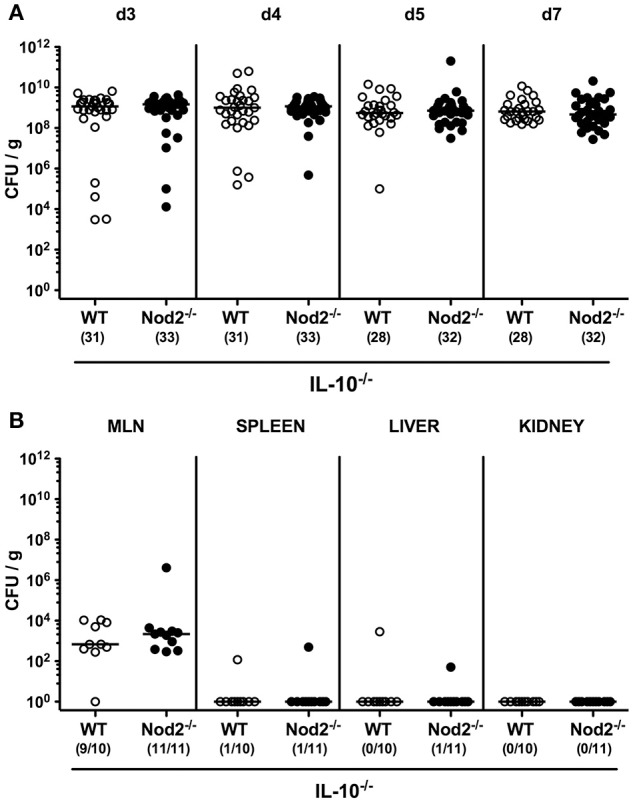
Intestinal colonization and translocation of *C. jejuni* strain 81–176 following peroral infection of secondary abiotic IL-10^−/−^ mice lacking Nod2. Secondary abiotic IL-10^−/−^ (WT IL-10^−/−^; white circles) and IL-10^−/−^ mice lacking Nod2 (Nod2^−/−^ IL-10^−/−^; black circles) were generated by broad-spectrum antibiotic treatment and perorally infected with *C. jejuni* strain 81–176 by gavage at day (d) 0 and d1. Pathogenic loads (CFU, colony forming units per gram) were assessed **(A)** in fecal samples over time post-infection as indicated by culture. **(B)** Pathogenic translocation to mesenteric lymph nodes (MLN) and extra-intestinal compartments including spleen, liver, and kidney were determined in organ homogenates of respective *ex vivo* biopsies (by culture). Medians (black bars) and numbers of mice harboring *C. jejuni* strain 81–176 out of the total number of analyzed animals are given in parentheses. Data were pooled from four **(A)** and two **(B)** independent experiments.

We next included secondary abiotic Nod2^−/−^ IL-10^−/−^ mice into our infection experiments. *C. jejuni* was able to stably colonize the intestines of both IL-10^−/−^ and Nod2^−/−^ IL-10^−/−^ mice until day 7 p.i. with high median densities of ~10^9^ CFU per gram feces (Figure 2A). Whereas, viable pathogens could be detected in ≥90% of MLN derived from infected mice, *C. jejuni* did virtually not translocate to extra-intestinal compartments such as spleen, liver and kidney at day 7 p.i. (Figure 2B). Hence, Nod2 did neither affect intestinal colonization nor translocation properties of *C. jejuni* in secondary abiotic IL-10^−/−^ mice.

### Colonic mucin-2 expression in secondary abiotic IL-10^−/−^ mice lacking Nod2

**Figure 3:**
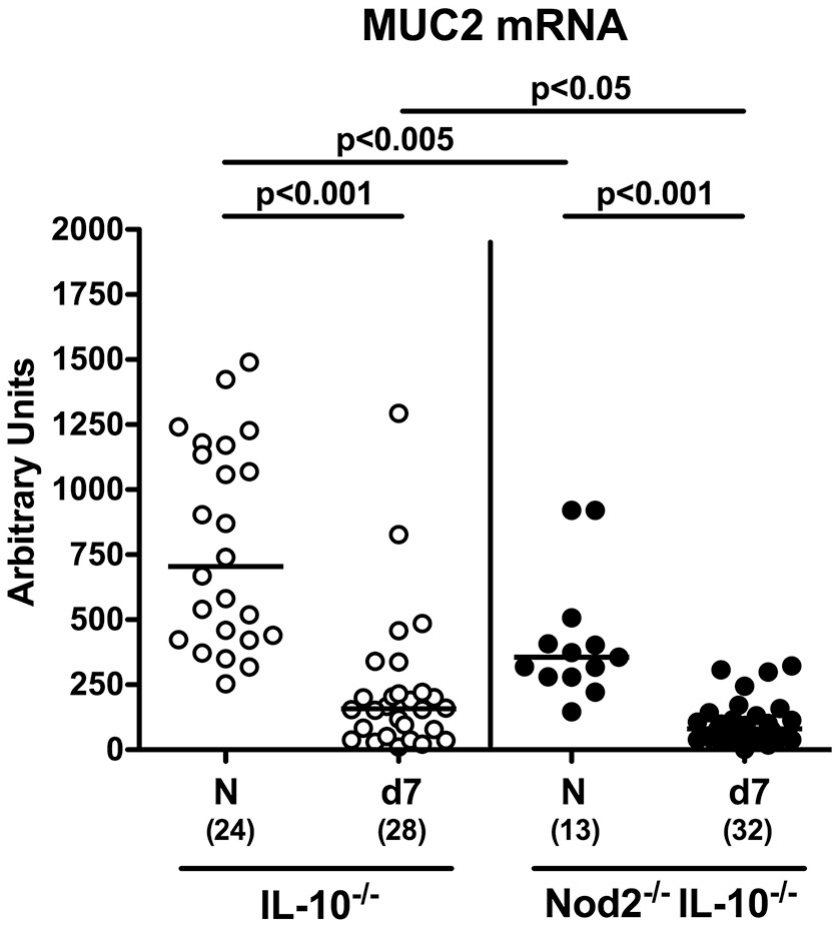
Colonic mucin-2 expression in *C. jejuni* strain 81–176 infected secondary abiotic IL-10^−/−^ mice lacking Nod2. Secondary abiotic IL-10^−/−^ (white circles) and IL-10^−/−^ mice lacking Nod2 (Nod2^−/−^ IL-10^−/−^; black circles) were generated by broad-spectrum antibiotic treatment and perorally infected with *C. jejuni* strain 81–176 by gavage at day (d) 0 and d1. Mucin-2 (MUC2) mRNA levels were determined in colonic *ex vivo* biopsies at day 7 post-infection by Real Time PCR and expressed as Arbitrary Units (fold expression). Naive (N) mice served as uninfected controls. Medians (black bars), levels of significance (*p*-values) determined by Mann–Whitney *U*-test and numbers of analyzed animals (in parentheses) are indicated. Data were pooled from four independent experiments.

Mucin-2 is known to constitute a key component of the mucus layer covering the intestinal epithelium and thereby combats bacterial infections and maintains epithelial barrier integrity (Velcich et al., 2002; McGuckin et al., 2011). We therefore addressed whether mucin-2 mRNA expression was affected in IL-10^−/−^ mice lacking Nod2. Already in the basal state, mucin-2 levels were lower in the colon of secondary abiotic Nod2^−/−^ IL-10^−/−^ mice as compared to IL-10^−/−^ counterparts (*p* < 0.005; Figure 3). At day 7 following *C. jejuni* strain 81–176 infection, colonic mucin-2 expression was down-regulated in mice of either genotype (*p* < 0.001), but even more distinctly in Nod2^−/−^ IL-10^−/−^ vs. IL-10^−/−^ mice (*p* < 0.05; Figure 3). Hence, Nod2 deficiency is associated with down-regulated colonic mucin-2 expression levels in secondary abiotic IL-10^−/−^ mice.

### Macroscopic sequelae of *C. jejuni* infection of secondary abiotic IL-10^−/−^ mice lacking Nod2

**Figure 4:**
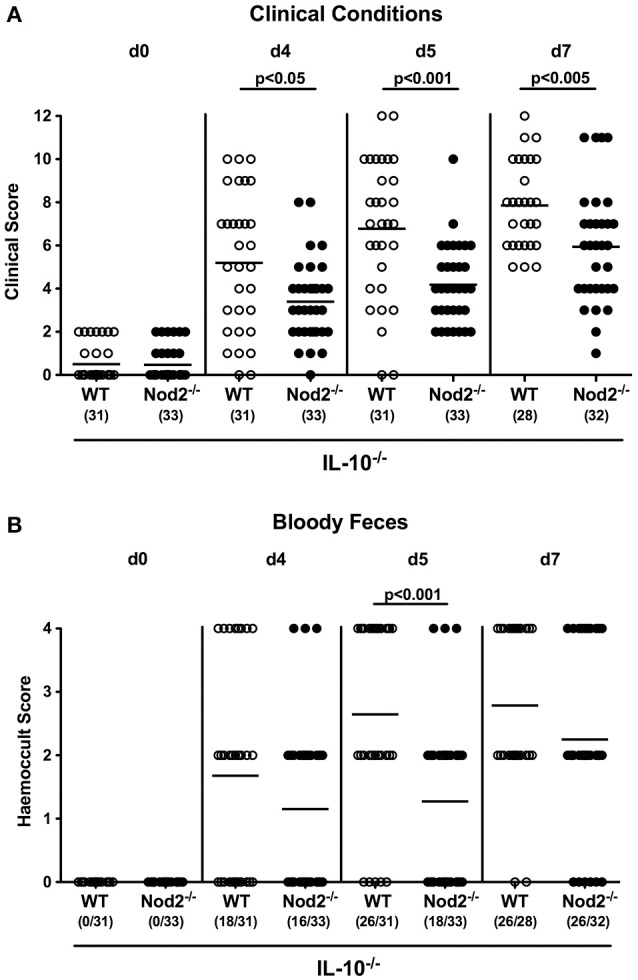
Clinical conditions in *C. jejuni* strain 81–176 infected secondary abiotic IL-10^−/−^ mice lacking Nod2. Secondary abiotic IL-10^−/−^ (WT IL-10^−/−^; white circles) and IL-10^−/−^ mice lacking Nod2 (Nod2^−/−^ IL-10^−/−^; black circles) were generated by broad-spectrum antibiotic treatment and perorally infected with *C. jejuni* strain 81–176 by gavage at day (d) 0 and d1. **(A)** Clinical symptoms and **(B)** occurrence of fecal blood were assessed before and after infection applying respective standardized clinical scoring systems (see Section Materials and Methods). Means (black bars), level of significance (*p*-values) determined by Mann–Whitney *U*-test and numbers of analyzed animals (in parentheses) are indicated. Data were pooled from four independent experiments.

We next assessed whether Nod2 deficiency had an impact on the clinical outcome upon *C. jejuni* infection of secondary abiotic IL-10^−/−^ mice. To address this, we applied a cumulative clinical scoring system assessing severities of clinical conditions and of diarrhea in particular, and the microscopic or even macroscopic abundance of blood in fecal samples. Within 48 h following the latest *C. jejuni* challenge (i.e., day 3 p.i.), clinical scores of infected mice had substantially increased irrespective of the genotype (*p* < 0.001; Figure S1) and were progressively rising thereafter (*p* < 0.05–0.001; Figure S1). Interestingly, IL-10^−/−^ mice lacking Nod2 were less compromised following *C. jejuni* infection than IL-10^−/−^ controls as indicated by lower cumulative clinical scores in the former at days 4, 5, and 7 p.i. (*p* < 0.05–0.001; Figure 4A). Furthermore, infected Nod2^−/−^ IL-10^−/−^ mice were suffering less distinctly from bloody diarrhea when compared to IL-10^−/−^ mice as indicated by lower haemoccult scores and less frequent abundance of blood in fecal samples in the former at day 5 p.i. (Figure 4B). Thus, Nod2 signaling worsens the clinical outcome in *C. jejuni* infected secondary abiotic IL-10^−/−^ mice.

### Microscopic sequelae of *C. jejuni* infection of secondary abiotic IL-10^−/−^ mice lacking Nod2

**Figure 5:**
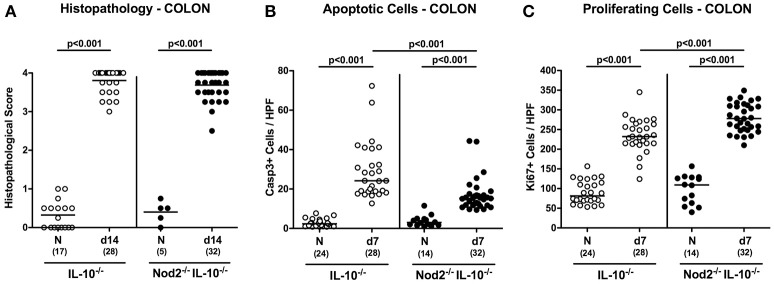
Microscopic sequelae in large intestines of *C. jejuni* strain 81–176 infected secondary abiotic IL-10^−/−^ mice lacking Nod2. Secondary abiotic IL-10^−/−^ (white circles) and IL-10^−/−^ mice lacking Nod2 (Nod2^−/−^ IL-10^−/−^; black circles) were generated by broad-spectrum antibiotic treatment and perorally infected with *C. jejuni* strain 81–176 by gavage at day (d) 0 and d1. **(A)** Histopathological mucosal changes were assessed in hematoxylin and eosin stained colonic paraffin sections. Furthermore, the average numbers of colonic epithelial **(B)** apoptotic cells (positive for caspase-3, Casp3) and **(C)** proliferating/regenerating cells (positive for Ki67) from six high power fields (HPF, 400x magnification) per animal were determined microscopically in immunohistochemically stained colonic paraffin sections at day 7 following *C. jejuni* infection. Naive (N) mice served as uninfected controls. Medians (black bars), levels of significance (*p*-values) determined by Mann–Whitney *U*-test and numbers of analyzed animals (in parentheses) are indicated. Data were pooled from four independent experiments.

We next assessed whether the better macroscopic outcome of *C. jejuni* infected secondary abiotic IL-10^−/−^ mice lacking Nod2 could also be observed on the microscopic level. At day 7 p.i. *C. jejuni* infected mice of either genotype exhibited comparable histopathological changes within the large intestinal mucosa and lamina propria that were indicative for acute ulcerative enterocolitis (n.s.; Figure 5A). Given that apoptosis is a well-established marker for the microscopic evaluation of intestinal inflammation including murine campylobacteriosis (Bereswill et al., 2011), we stained colonic paraffin section with caspase-3 antibodies by *in situ* immunohistochemistry. Upon *C. jejuni* infection, secondary abiotic mice of either genotype exhibited a multifold increase of apoptotic colonic epithelial cells (*p* < 0.001; Figure 5B). This increase, however, was less pronounced in Nod2^−/−^ IL-10^−/−^ as compared to IL-10^−/−^ mice at day 7 p.i. (*p* < 0.001; Figure 5B). We additionally stained colonic paraffin sections with Ki67 antibodies to quantitatively assess proliferating cells that were potentially counteracting apoptotic responses upon *C. jejuni* infection. Until day 7 p.i. Ki67 positive cell numbers increased multifold in the colonic epithelia of Nod2^−/−^ IL-10^−/−^ as well as of IL-10^−/−^ mice (*p* < 0.001; Figure 5C), but more distinctly in the former (*p* < 0.001; Figure 5C). Taken together, the better clinical outcome observed in *C. jejuni* infected IL-10^−/−^ mice lacking Nod2 was supported by less distinct apoptotic and higher proliferating / regenerative responses in colonic epithelia.

### Colonic immune cell responses in *C. jejuni* infected secondary abiotic IL-10^−/−^ mice lacking Nod2

**Figure 6:**
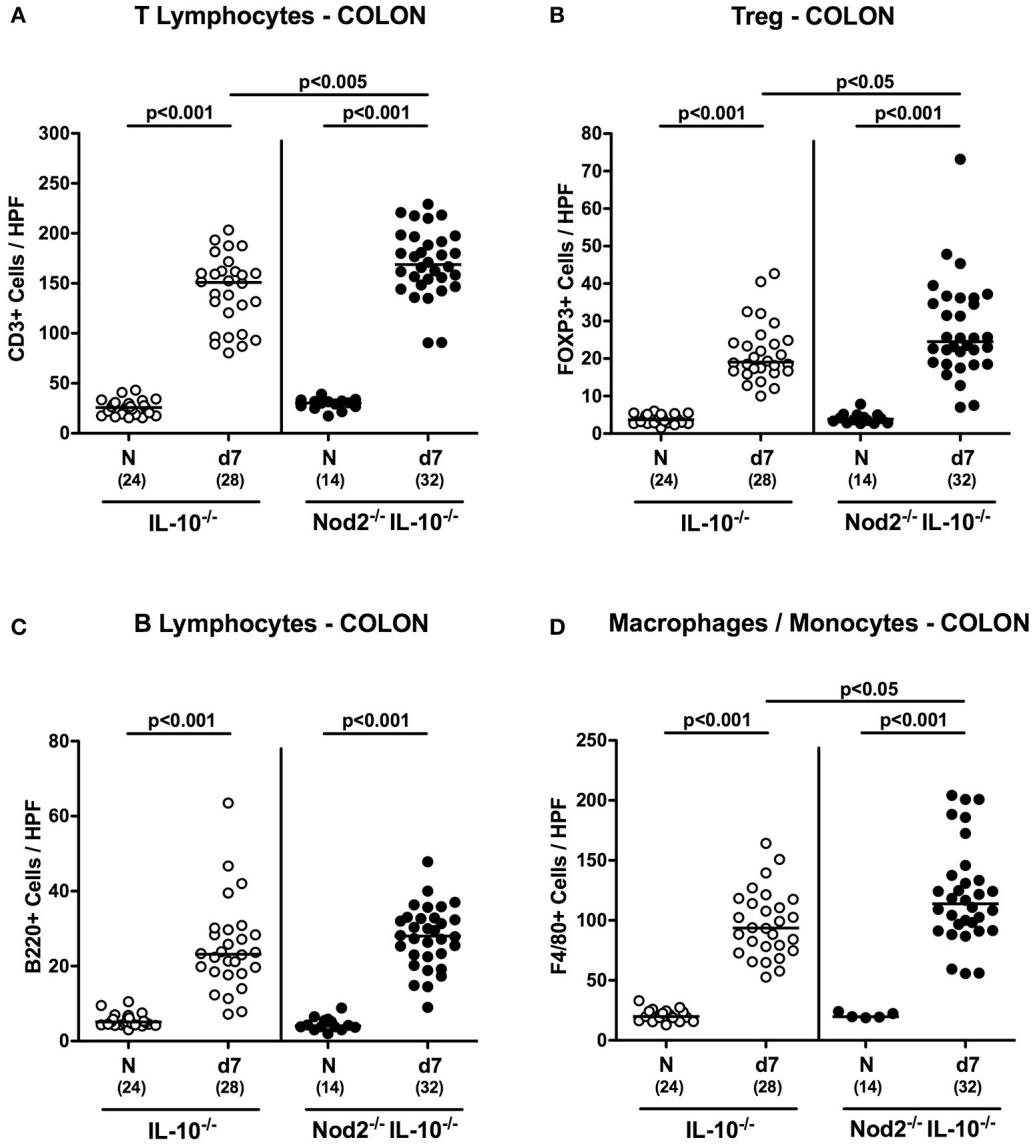
Colonic immune cell responses in *C. jejuni* strain 81–176 infected secondary abiotic IL-10^−/−^ mice lacking Nod2. Secondary abiotic IL-10^−/−^ (white circles) and IL-10^−/−^ mice lacking Nod2 (Nod2^−/−^ IL-10^−/−^; black circles) were generated by broad-spectrum antibiotic treatment and perorally infected with *C. jejuni* strain 81–176 by gavage at day (d) 0 and d1. The average numbers of colonic epithelial **(A)** T lymphocytes (positive for CD3), **(B)** regulatory T cells (Treg; positive for FOXP3), **(C)** B lymphocytes (positive for B220), and **(D)** macrophages and monocytes (positive for F4/80) from six high power fields (HPF, 400x magnification) per animal were determined microscopically in immunohistochemically stained colonic paraffin sections at day 7 following *C. jejuni* infection. Naive (N) mice served as uninfected controls. Medians (black bars), levels of significance (*p*-values) determined by Mann–Whitney *U*-test and numbers of analyzed animals (in parentheses) are indicated. Data were pooled from four independent experiments.

Recruitment of pro-inflammatory immune cells to the site of infection is a known key feature of intestinal inflammation including campylobacteriosis (Bereswill et al., 2011; Alutis et al., 2015a). We therefore quantitatively assessed distinct innate and adaptive immune cell populations in the large intestinal mucosa and lamina propria of infected mice by *in situ* immunohistochemistry. Seven days following *C. jejuni* infection numbers of adaptive immune cells including T and B lymphocytes as well as regulatory T cells (Treg) increased multifold in the large intestines of secondary abiotic mice of either genotype (*p* < 0.001; Figures 6A–C). These increases were also true for innate immune cell populations such as macrophages and monocytes (*p* < 0.001; Figure 6D). The observed increases in T lymphocytes and Treg as well as in macrophages and monocytes were more pronounced in IL-10^−/−^ mice lacking Nod2 as compared to IL-10^−/−^ controls at day 7 p.i. (*p* < 0.05–0.005; Figures 6A,B,D). Hence, whereas macroscopic and microscopic outcomes of *C. jejuni* infection was more favorable in secondary abiotic IL-10^−/−^ mice with Nod2 deficiency, innate as well as adaptive immune cells were even more abundant in large intestines of infected Nod2^−/−^ IL-10^−/−^ mice.

### Colonic cytokine responses in *C. jejuni* infected secondary abiotic IL-10^−/−^ mice lacking Nod2

**Figure 7:**
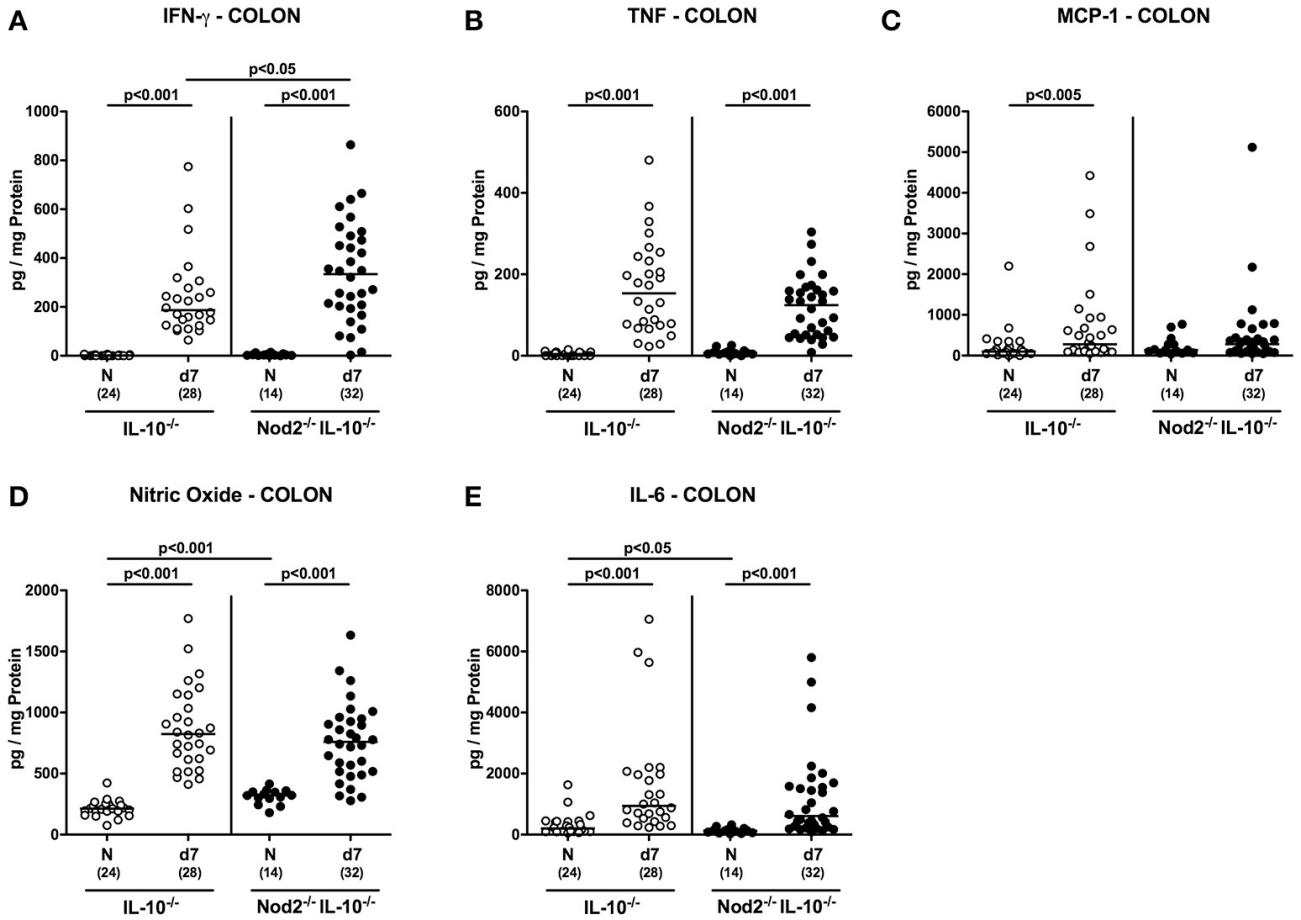
Colonic secretion of pro-inflammatory mediators in *C. jejuni* strain 81–176 infected secondary abiotic IL-10^−/−^ mice lacking Nod2. Secondary abiotic IL-10^−/−^ (white circles) and IL-10^−/−^ mice lacking Nod2 (Nod2^−/−^ IL-10^−/−^; black circles) were generated by broad-spectrum antibiotic treatment and perorally infected with *C. jejuni* strain 81–176 by gavage at day (d) 0 and d1. **(A)** IFN-γ, **(B)** TNF, **(C)** MCP-1, **(D)** nitric oxide, and **(E)** IL-6 concentrations were determined in supernatants of colonic *ex vivo* biopsies at day 7 post-infection. Naive (N) mice served as uninfected controls. Medians (black bars), level of significance (*p*-value) determined by Mann–Whitney *U*-test and numbers of analyzed animals (in parentheses) are indicated. Data were pooled from four independent experiments.

We next measured secretion of pro-inflammatory mediators in supernatants of colonic *ex vivo* biopsies. Irrespective of the genotype, IFN-γ, TNF, nitric oxide, and IL-6 concentrations increased in large intestines of secondary abiotic mice until day 7 p.i. (*p* < 0.001; Figures 7A,B,D,E). Colonic IFN-γ concentrations were, however, higher in Nod2^−/−^ IL-10^−/−^ as compared to IL-10^−/−^ mice at day 7 p.i. (*p* < 0.05; Figure 7A). Moreover, MCP-1 levels were elevated in the colon of infected IL-10^−/−^ mice (*p* < 0.05; Figure 7C) and showed a trend toward increased concentrations also in Nod2^−/−^ IL-10^−/−^ mice (not significant due to high standard deviations). Notably, basal nitric oxide levels were higher in naive IL-10^−/−^ mice lacking Nod2 than in IL-10^−/−^ controls (*p* < 0.001; Figure 7D). Hence, higher abundances of innate and adaptive immune cell populations in secondary abiotic IL-10^−/−^ mice with Nod2 deficiency were accompanied by higher colonic IFN-γ secretion.

We next assessed mRNA expression levels of pro-inflammatory cyokines in colonic *ex vivo* biopsies. As on protein level, large intestinal IFN-γ and TNF mRNA were up-regulated upon *C. jejuni* infection (*p* < 0.001; Figures S2A,B). IFN-γ mRNA levels were, however, higher in the colon of Nod2^−/−^ IL-10^−/−^ as compared to IL-10^−/−^ mice at day 7 p.i. (*p* < 0.05; Figure S2A). In addition, IL-17A and IL-1β mRNA were up-regulated in colonic *ex vivo* biopsies irrespective of the genotypes of mice (*p* < 0.001; Figures S2C,D).

**Figure 8:**
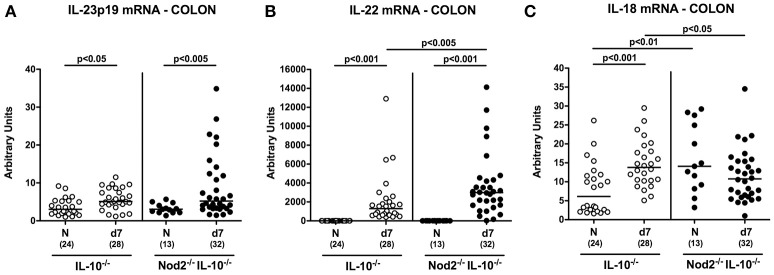
Colonic mRNA expression of IL-23p19, IL-22, and IL-18 in *C. jejuni* strain 81–176 infected secondary abiotic IL-10^−/−^ mice lacking Nod2. Secondary abiotic IL-10^−/−^ (white circles) and IL-10^−/−^ mice lacking Nod2 (Nod2^−/−^ IL-10^−/−^; black circles) were generated by broad-spectrum antibiotic treatment and perorally infected with *C. jejuni* strain 81–176 by gavage at day (d) 0 and d1. Expression of **(A)** IL-23p19, **(B)** IL-22, and **(C)** IL-18 mRNA were determined in colonic *ex vivo* biopsies at day 7 post-infection by Real Time PCR and expressed as Arbitrary Units (fold expression). Naive (N) mice served as uninfected controls. Medians (black bars), level of significance (*p*-value) determined by Mann–Whitney *U*-test and numbers of analyzed animals (in parentheses) are indicated. Data were pooled from four independent experiments.

We have recently shown that the IL-23/IL-22/IL-18 axis mediates *C. jejuni* infection *in vivo* (Alutis et al., 2015a; Bereswill et al., 2016; Heimesaat et al., 2016a,b) and therefore determined respective cytokine expression levels in colonic *ex vivo* biopsies. At day 7 p.i. IL-23p19 and IL-22 mRNA were up-regulated in the large intestines of both Nod2^−/−^ IL-10^−/−^ and IL-10^−/−^ control mice (*p* < 0.05–0.001; Figures 8A,B), whereas *C. jejuni* induced increased IL-18 mRNA levels could be measured in the latter only (*p* < 0.001; Figure 8C). *C. jejuni* infected Nod2^−/−^ IL-10^−/−^ mice exhibited higher anti-inflammatory IL-22 (*p* < 0.005; Figure 8B), but lower IL-18 mRNA levels in their large intestines as compared to IL-10^−/−^ controls (*p* < 0.05; Figure 8C).

### Pro-inflammatory cytokine responses in mesenteric lymph nodes and spleens of *C. jejuni* infected secondary abiotic IL-10^−/−^ mice lacking Nod2

**Figure 9:**
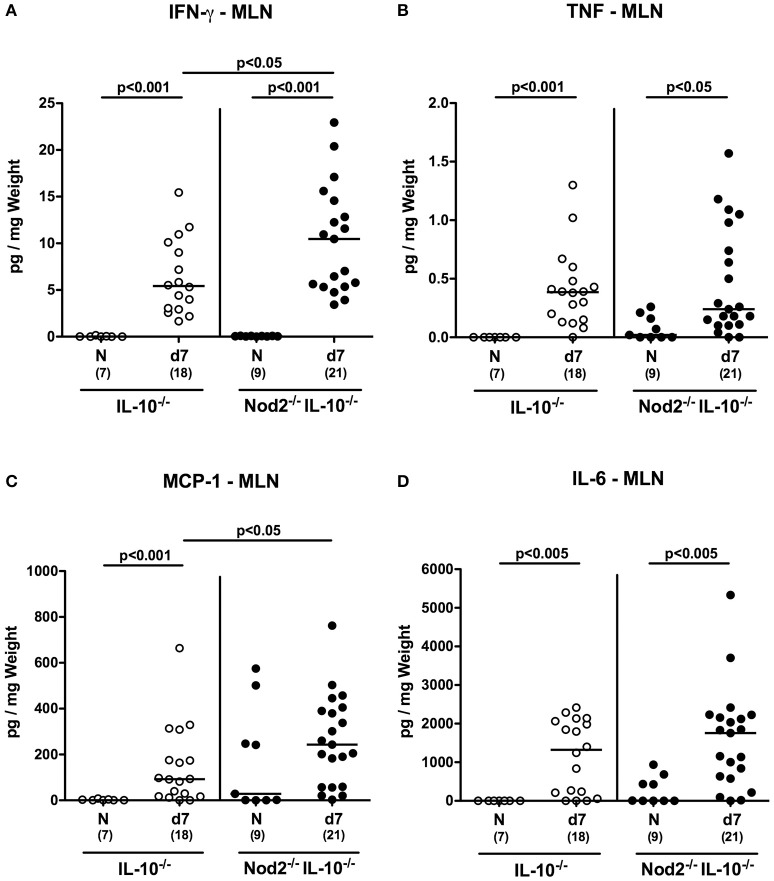
Secretion of pro-inflammatory cytokines in mesenteric lymph nodes of *C. jejuni* strain 81–176 infected secondary abiotic IL-10^−/−^ mice lacking Nod2. Secondary abiotic IL-10^−/−^ (white circles) and IL-10^−/−^ mice lacking Nod2 (Nod2^−/−^ IL-10^−/−^; black circles) were generated by broad-spectrum antibiotic treatment and perorally infected with *C. jejuni* strain 81–176 by gavage at day (d) 0 and d1. **(A)** IFN-γ, **(B)** TNF, **(C)** MCP-1, and **(D)** IL-6 concentrations were determined in supernatants of *ex vivo* biopsies derived from mesenteric lymph nodes (MLN) at day 7 post-infection. Naive (N) mice served as uninfected controls. Medians (black bars), level of significance (*p*-value) determined by Mann–Whitney *U*-test and numbers of analyzed animals (in parentheses) are indicated. Data were pooled from three independent experiments.

We next assessed pro-inflammatory cytokine secretion in intestinal draining and systemic lymphatic compartments, namely MLN and spleen, respectively, of *C. jejuni* infected secondary abiotic IL-10^−/−^ mice lacking Nod2. At day 7 p.i. increased IFN-γ, TNF, and IL-6 concentrations were measured in supernatants of MLN taken from secondary abiotic mice of either genotype (*p* < 0.05–0.001; Figures 9A,B,D). Increased MCP-1 levels were determined in MLN of *C. jejuni* infected IL-10^−/−^ mice, whereas Nod2^−/−^ IL-10^−/−^ animals displayed a trend toward higher MCP-1 concentrations as compared to naive mice (n.s.; Figure 9C).

**Figure 10:**
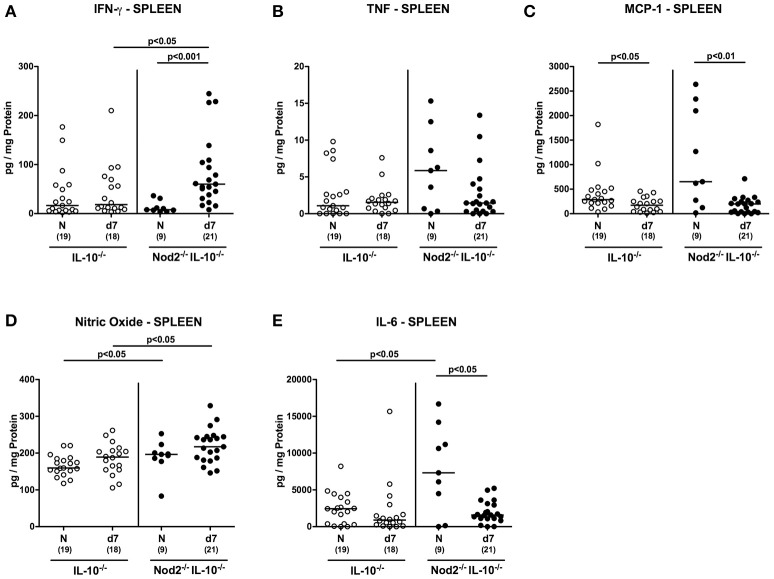
Splenic secretion of pro-inflammatory mediators in *C. jejuni* strain 81–176 infected secondary abiotic IL-10^−/−^ mice lacking Nod2. Secondary abiotic IL-10^−/−^ (white circles) and IL-10^−/−^ mice lacking Nod2 (Nod2^−/−^ IL-10^−/−^; black circles) were generated by broad-spectrum antibiotic treatment and perorally infected with *C. jejuni* strain 81–176 by gavage at day (d) 0 and d1. **(A)** IFN-γ, **(B)** TNF, **(C)** MCP-1, **(D)** nitric oxide, and **(E)** IL-6 concentrations were determined in supernatants of *ex vivo* biopsies derived from spleens at day 7 postinfection. Naive (N) mice served as uninfected controls. Medians (black bars), level of significance (*p*-value) determined by Mann–Whitney *U*-test and numbers of analyzed animals (in parentheses) are indicated. Data were pooled from three independent experiments.

In spleens, IFN-γ concentrations increased upon *C. jejuni* infection of Nod2^−/−^ IL-10^−/−^ mice only (*p* < 0.001; Figure 10A) and were higher as compared to IL-10^−/−^ counterparts at day 7 p.i. (*p* < 0.05; Figure 10A). In addition, splenic nitric oxide concentrations were higher in infected IL-10^−/−^ mice lacking Nod2 as compared to IL-10^−/−^ controls (*p* < 0.05; Figure 10D). Upon *C. jejuni* infection, splenic MCP-1 levels decreased in secondary abiotic mice irrespective of the genotype (*p* < 0.05–0.01; Figure 10C), whereas IL-6 secretion was less distinct in Nod2^−/−^ IL-10^−/−^ only at day 7 p.i. as compared to naive controls (*p* < 0.05; Figure 10E). In line with splenic IL-6 results, at least a trend toward lower MCP-1 concentrations could be observed in spleens of infected Nod2^−/−^ IL-10^−/−^ mice as compared to naive counterparts (n.s.; Figure 10B). Notably, basal IL-6 levels were higher in spleens of Nod2^−/−^ IL-10^−/−^ than IL-10^−/−^ control mice (*p* < 0.05; Figure 10E).

Hence, the increases in pro-inflammatory cytokine secretion observed in large intestines of *C. jejuni* infected secondary abiotic mice were supported by results derived from MLN. If compared to IL-10^−/−^ controls, secondary abiotic IL-10^−/−^ mice lacking Nod2 exhibited higher IFN-γ concentrations in intestinal compartments such as colon and MLN as well as in extra-intestinal/systemic sites (i.e., spleen) at day 7 p.i.

## Discussion

Given that host innate immune responses are pivotal for combating enteropathogenic infections including campylobacteriosis, we here investigated the impact of Nod2 during *C. jejuni* infection of secondary abiotic mice lacking IL-10^−/−^. Notably, 1 week following *C. jejuni* infection colonic Nod2 mRNA expression was down-regulated in large intestines of both secondary abiotic WT and IL-10^−/−^ mice, whereas Nod2 did not affect gastrointestinal colonization of *C. jejuni*. This is well in line with our very recent investigations in conventionally colonized Nod2^−/−^ mice displaying comparable pathogenic loads in their gastrointestinal tract (Bereswill et al., 2017). One might have expected higher intestinal *C. jejuni* loads in Nod2 deficient IL-10^−/−^ mice, given that Nod2 deficiency was shown to be associated with compromised expression of antimicrobioal peptides including defensins leading to insufficient clearance of the pathogen by the host (Huttner and Bevins, 1999; Kobayashi et al., 2005). In fact, Nod2^−/−^ mice have been shown to be more susceptible to infection with other enteropathogens such as Salmonella Typhimurium, *Yersinia pseudotuberculosis*, or *Listeria monocytogenes* (Kobayashi et al., 2005; Meinzer et al., 2008). Notably, in our study *C. jejuni* infection induced a down-regulation of colonic mucin-2 mRNA that constitutes an integral part of the mucus layer covering the intestinal epithelium, thereby providing epithelial barrier integrity and preventing the host from bacterial species invading from the intestinal lumen (Velcich et al., 2002; McGuckin et al., 2011). Remarkably, mucin-2 mRNA expression was Nod2 dependent and even more distinctly down-regulated in the large intestines of *C. jejuni* infected Nod2^−/−^ IL-10^−/−^ mice as compared to control mice in our study which was also the case under basal (i.e., naive, uninfected) conditions. Like pathogenic colonization, however, translocation of viable *C. jejuni* from the intestinal lumen to extra-intestinal and systemic compartments occurred Nod2 independently. In fact, viable bacteria could be isolated from liver, kidney and spleen of mice irrespective of their genotype in single cases only. Despite lack of bacterial translocation to systemic sites, however, increased IFN-γ levels could not only be detected in the colon and MLN, but also in the spleen of *C. jejuni* infected Nod2^−/−^ IL-10^−/−^ and IL-10^−/−^ mice that were higher in the former. It is tempting to speculate that elevated systemic levels were rather due to circulating *C. jejuni* cell wall constituents such as lipooligosaccharide or other Toll-like-receptor (TLR) ligands. Conversely, *C. jejuni* infection resulted in decreased splenic secretion of MCP-1 and IL-6 upon *C. jejuni* infection, whereas respective cytokine levels were elevated in intestinal compartments including colon and MLN. This might be explained by recruitment of innate and adaptive immune cells from the spleen to the site of infection as supported by increased numbers of T lymphocytes as well as of macrophages and monocytes in the mucosa and lamina propria of *C. jejuni* infected mice of either genotype. In support, we could demonstrate previously that colonic T cells numbers were higher in *C. jejuni* infected conventional Nod2^−/−^ as compared to WT mice (Bereswill et al., 2017).

Very recently, our group elucidated the role of the IL-23/IL-22/IL-18 axis in murine host -*C. jejuni* interaction (Alutis et al., 2015b; Bereswill et al., 2016; Heimesaat et al., 2016a,b). In line with our studies in secondary abiotic WT mice (Alutis et al., 2015b), *C. jejuni* infection induced an up-regulation of IL-23p19, IL-22, and IL-18 mRNA in large intestines of secondary abiotic IL-10^−/−^ mice as shown here. In support, Malik et al. reported increased colonic IL-22 mRNA levels in *C. jejuni* infected conventionally colonized IL-10^−/−^ mice (Malik et al., 2014). IL-22, as member of the IL-10 cytokine family, can have both pro- and anti-inflammatory properties, depending on the respective intestinal tissue, immunological prerequisites, and the surrounding cytokine milieu (Eidenschenk et al., 2014; Heimesaat et al., 2016b). Whereas, in the small intestines IL-22 exerts pro-inflammatory properties (Munoz et al., 2009, 2011, 2015), IL-22 has anti-inflammatory functions in the colon (Eidenschenk et al., 2014) and proven effective in anti-microbial host defense against *C. jejuni* (Bereswill et al., 2016; Heimesaat et al., 2016b). In the present study, *C. jejuni* induced up-regulation of anti-inflammatory IL-22 mRNA in the colon was even more pronounced in Nod2 deficient IL-10^−/−^ as compared to IL-10^−/−^ counterparts. Conversely, large intestinal mRNA levels of IL-18 that is known to amplify IL-22 expression during intestinal inflammation (Munoz et al., 2015) were lower in Nod2^−/−^ IL-10^−/−^ vs. IL-10^−/−^ controls, which might have been due to a potential negative feedback loop between IL-22 and IL-18.

Despite elevated large intestinal innate and adaptive immune cell influx (of macrophages/monocytes and T lymphocytes, respectively) and increased IFN-γ concentrations in the colon, MLN and spleen following *C. jejuni* infection, Nod2^−/−^ IL-10^−/−^ were not only less compromised from their clinical aspect, but also displayed less distinct apoptotic colonic epithelial cell responses than IL-10^−/−^ controls. These observed and presumably unexpected effects might result from effective counter-regulatory responses such as higher Treg numbers and anti-inflammatory IL-22 expression levels in the large intestines of *C. jejuni* infected Nod2 deficient IL-10^−/−^ mice as compared to IL-10^−/−^ controls that were accompanied by higher numbers of Ki67+ colonic epithelial cells in the former indicative for accelerated regenerative measures of the colonic epithelium counter-acting *C. jejuni* induced cell damage. Hence, Nod2 might exert both pro-and anti-inflammatory functions in the complex interplay of innate and adaptive immunity with enteropathogens such as *C. jejuni*.

To date, experimental data regarding the distinct role of Nod2 in intestinal inflammation are inconclusive. Depending on the applied *in vivo* model, Nod2 deficiency might either enhance or even prevent from colitis development. Less severe chronic colitis could be observed following adoptive transfer of Nod2^−/−^ T cells into immunocompromised mice, for instance, indicating that Nod2 signaling exacerbates large intestinal immunopathology (Shaw et al., 2008). Whereas, MDP application could prevent from 2,4,6-trinitrobenzenesulphonic acid (TNBS) colitis, preventive properties of MDP were abrogated in Nod2 deficient mice indicative for a protective role of Nod2 signaling (Watanabe et al., 2008). In another study, however, Nod2 was shown to rather promote colitis, given that Nod2 deficient IL-10^−/−^ mice were protected from large intestinal inflammation (Jamontt et al., 2013). Conflicting data were derived from with antibiotics pretreated Nod2^−/−^ IL-10^−/−^ mice that displayed accelerated colitis following *C. jejuni* infection indicating that Nod2 was essential for controlling murine campylobacteriosis (Sun and Jobin, 2014). In this elegant study, mice were pretreated with an antibiotic cocktail for 7 days and followed up for 21 days upon *C. jejuni* infection. Hence, our discrepant results reported here might be most likely due to substantial differences in experimental set-ups. Given that conventional IL-10^−/−^ mice develop chronic colitis due to antigenic stimuli derived from their commensal intestinal microbiota (Haag et al., 2012), we subjected mice immediately after weaning by the age of 3 weeks to broad-spectrum antibiotic treatment in order to eradicate potential colitogenic stimuli from their microbiota. Following a much longer course of broad-spectrum antibiotic treatment (i.e., 8 weeks) with a different, quintuple antibiotic regimen, secondary abiotic IL-10^−/−^ mice develop severe ulcerative enterocolitis with bloody diarrhea that is not self-limiting and requires necrospsy until day 7 p.i. (Haag et al., 2012; Heimesaat et al., 2014a,c; Fiebiger et al., 2016). Hence, in our study we surveyed *C. jejuni*-host interactions to a much earlier time point (i.e., 1 week p.i.) than Sun and Jobin did (i.e. 3 weeks p.i.). Our group could further show that Nod2 protected mice from *Toxoplasma gondii* induced acute ileitis (Heimesaat et al., 2014b). Overall, inconclusive results might be due to differences in the applied *in vivo* models and fundamental discrepancies in experimental setups as well as due to potential dichotomous functions of Nod2.

We conclude that Nod2 signaling is required for the fine-tuned innate and adaptive local (i.e., intestinal) and systemic immune responses upon *C. jejuni* infection of secondary abiotic IL-10^−/−^ mice, but does not limit pathogenic infection. Further studies are needed to unravel the distinct regulatory mechanisms combating campylobacteriosis.

## Author contributions

MH: Designed and performed experiments, analyzed data, wrote paper; UG: Performed experiments, analyzed data; MA: Performed experiments, analyzed data; AF: Performed experiments, analyzed data; SB: Provided advice in experimental design, critically discussed results, co-edited paper.

### Conflict of interest statement

The authors declare that the research was conducted in the absence of any commercial or financial relationships that could be construed as a potential conflict of interest.

## Supplementary material

Figure S1 Kinetic survey of clinical conditions of secondary abiotic IL-10^−/−^ mice lacking Nod2 following *C. jejuni* strain 81–176 infection. Secondary abiotic **(A)** IL-10^−/−^ (white circles) and **(B)** IL-10^−/−^ mice lacking Nod2 (Nod2^−/−^ IL-10^−/−^; black circles) were generated by broad-spectrum antibiotic treatment and perorally infected with *C. jejuni* strain 81–176 by gavage at day (d) 0 and d1. Severities of clinical symptoms before and after infection were quantitatively assessed applying a standardized clinical score (see Section Materials and Methods). Means (black bars) and levels of significance (*p*-values) determined by Mann–Whitney *U*-test are indicated. Numbers of analyzed mice are given in parentheses. Data were pooled from four independent experiments.

Figure S2 Colonic mRNA expression of pro-inflammatory cytokines in *C. jejuni* strain 81–176 infected secondary abiotic IL-10^−/−^ mice lacking Nod2. Secondary abiotic IL-10^−/−^ (white circles) and IL-10^−/−^ mice lacking Nod2 (Nod2^−/−^ IL-10^−/−^; black circles) were generated by broad-spectrum antibiotic treatment and perorally infected with *C. jejuni* strain 81–176 by gavage at day (d) 0 and d1. Expression of **(A)** IFN-γ, **(B)** TNF, **(C)** IL-17A, and **(D)** IL-1β mRNA were determined in colonic *ex vivo* biopsies at day 7 post-infection by Real Time PCR and expressed as Arbitrary Units (fold expression). Naive (N) mice served as uninfected controls. Medians (black bars), level of significance (*p*-value) determined by Mann–Whitney *U*-test and numbers of analyzed animals (in parentheses) are indicated. Data were pooled from four independent experiments.

## Abbreviations

CBA: Cytometric Beat Assay
CFU: colony-forming units
H&E: hematoxylin and eosin
HPRT: hypoxanthine-phosphoribosyltransferase
MDP: muramyl dipeptide
MLN: mesenetric lymph nodes
MUC2: mucin-2
NO: nitric oxide
Nod: nucleotide-oligomerization-domain
PBS: phosphate buffered saline
p.i: postinfection
SPF: special pathogen free
TNBS: 2,4,6-trinitrobenzenesulphonic acid
TLR: Toll-like receptor
Treg: regulatory T cells
WT: wildtype.
