# Supplementary figures and images for: Absence of Nucleotide-Oligomerization-Domain-2 Is Associated with Less Distinct Disease in Campylobacter jejuni Infected Secondary Abiotic IL-10 Deficient Mice
Source: Front Cell Infect Microbiol. 2017 Jul 13;7:322. doi: 10.3389/fcimb.2017.00322 (PMC5508002; doi:10.3389/fcimb.2017.00322)

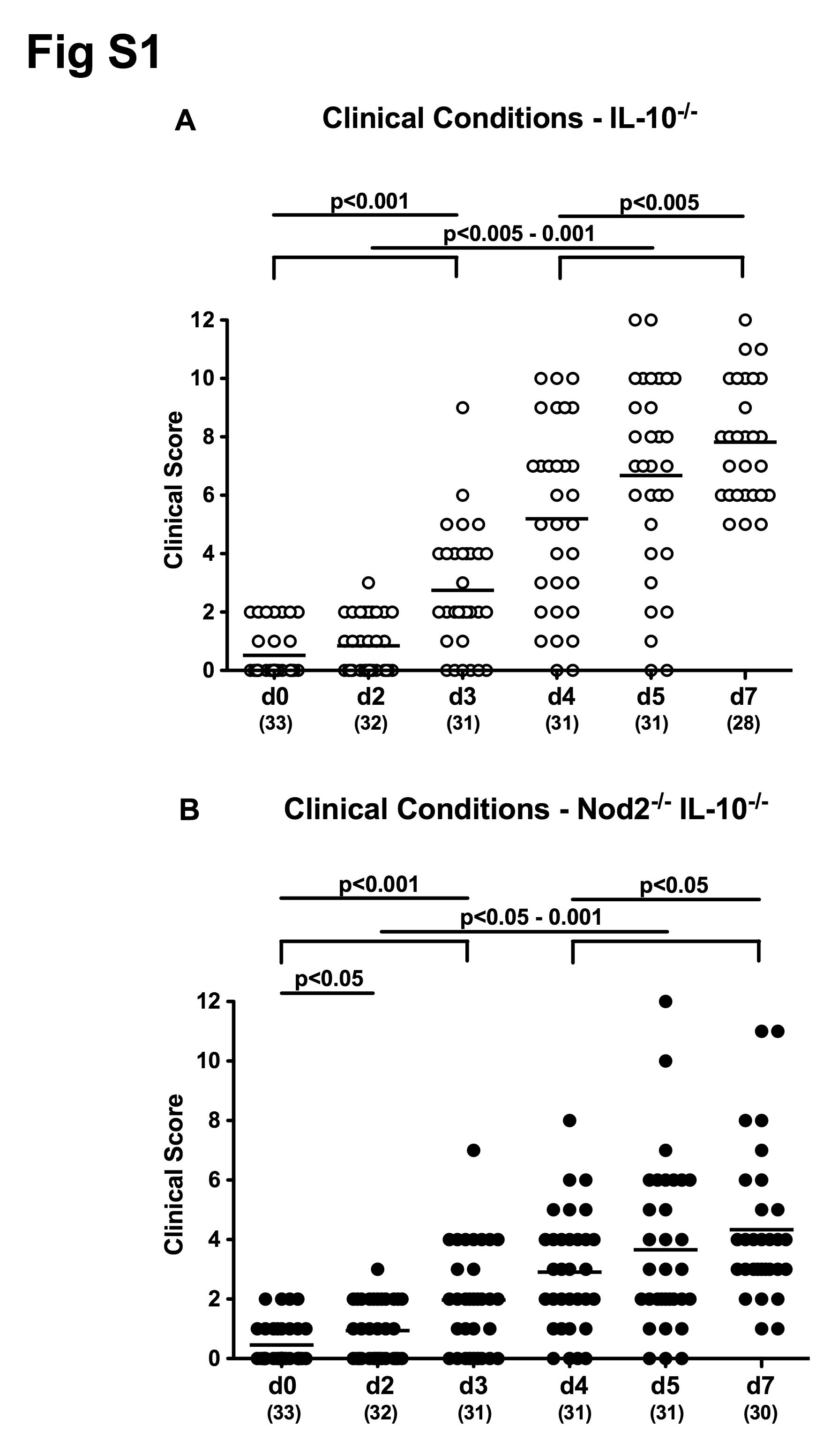

Supplement: Figure S1 — Kinetic survey of clinical conditions of secondary abiotic IL-10−/− mice lacking Nod2 following C. jejuni strain 81–176 infection. Secondary abiotic (A) IL-10−/− (white circles) and (B) IL-10−/− mice lacking Nod2 (Nod2−/− IL-10−/−; black circles) were generated by broad-spectrum antibiotic treatment and perorally infected with C. jejuni strain 81–176 by gavage at day (d) 0 and d1. Severities of clinical symptoms before and after infection were quantitatively assessed applying a standardized clinical score (see Section Materials and Methods). Means (black bars) and levels of significance (p-values) determined by Mann–Whitney U-test are indicated. Numbers of analyzed mice are given in parentheses. Data were pooled from four independent experiments. [file Image1.JPEG]

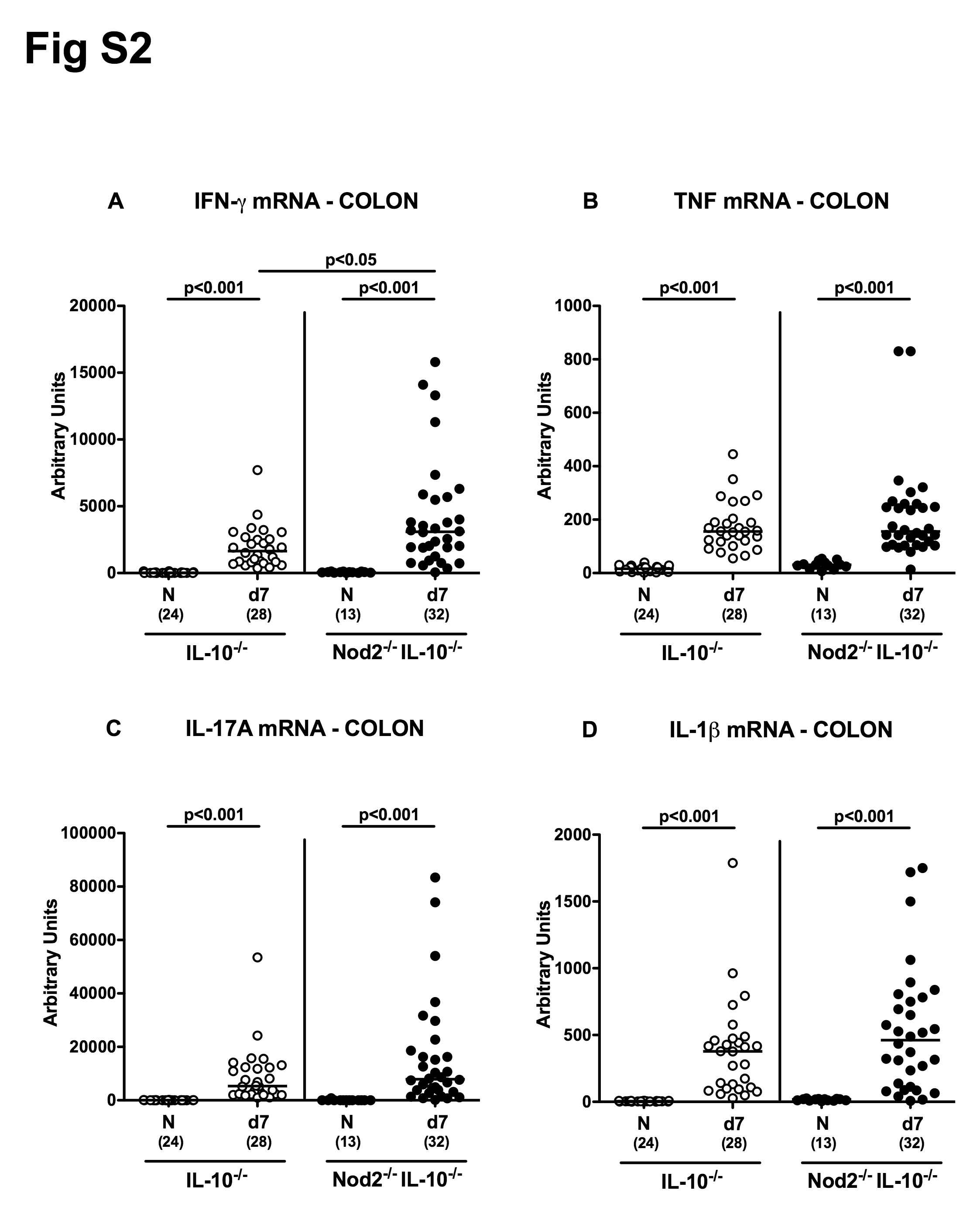

Supplement: Figure S2 — Colonic mRNA expression of pro-inflammatory cytokines in C. jejuni strain 81–176 infected secondary abiotic IL-10−/− mice lacking Nod2. Secondary abiotic IL-10−/− (white circles) and IL-10−/− mice lacking Nod2 (Nod2−/− IL-10−/−; black circles) were generated by broad-spectrum antibiotic treatment and perorally infected with C. jejuni strain 81–176 by gavage at day (d) 0 and d1. Expression of (A) IFN-γ, (B) TNF, (C) IL-17A, and (D) IL-1β mRNA were determined in colonic ex vivo biopsies at day 7 post-infection by Real Time PCR and expressed as Arbitrary Units (fold expression). Naive (N) mice served as uninfected controls. Medians (black bars), level of significance (p-value) determined by Mann–Whitney U-test and numbers of analyzed animals (in parentheses) are indicated. Data were pooled from four independent experiments. [file Image2.JPEG]
